# Complete Genome Sequence of *Pelosinus* sp. Strain UFO1 Assembled Using Single-Molecule Real-Time DNA Sequencing Technology

**DOI:** 10.1128/genomeA.00881-14

**Published:** 2014-09-04

**Authors:** Steven D. Brown, Sagar M. Utturkar, Timothy S. Magnuson, Allison E. Ray, Farris L. Poole, W. Andrew Lancaster, Michael P. Thorgersen, Michael W. W. Adams, Dwayne A. Elias

**Affiliations:** a Biosciences Division, Oak Ridge National Laboratory, Oak Ridge, Tennessee, USA; b Graduate School of Genome Science and Technology, University of Tennessee, Knoxville, Tennessee, USA; c Department of Biological Sciences, Idaho State University, Pocatello, Idaho, USA; d Biological Systems Department, Idaho National Laboratory, Idaho Falls, Idaho, USA; e Department of Biochemistry and Molecular Biology, University of Georgia, Athens, Georgia, USA

## Abstract

*Pelosinus* species can reduce metals such as Fe(III), U(VI), and Cr(VI) and have been isolated from diverse geographical regions. Five draft genome sequences have been published. We report the complete genome sequence for *Pelosinus* sp. strain UFO1 using only PacBio DNA sequence data and without manual finishing.

## GENOME ANNOUNCEMENT

*Pelosinus fermentans* strain R7 was isolated from Russian kaolin clays as the type strain and it can reduce Fe(III) during fermentative growth (1). Draft genome sequences for *P. fermentans* R7 and four strains from Hanford, Washington, USA, have been published (2–4). The *P. fermentans* 16S rRNA sequence dominated the lactate-based enrichment cultures from three geochemically contrasting soils from the Melton Branch Watershed, Oak Ridge, Tennessee, USA (5) and also at another stimulated, uranium-contaminated field site near Oak Ridge (6). For the current work, strain UFO1 was isolated from pristine sediments at a background field site in Oak Ridge and characterized as facilitating U(VI) reduction and precipitation with phosphate (7).

Earlier draft genome sequences of *Pelosinus* spp. were generated using a combination of Illumina and 454 technologies (3) or by Illumina sequencing alone (2, 4). Draft genome sizes ranged from 4.9 to 5.3 Mb, had G+C genome contents from 39.4 to 39.8%, and consisted of 65 to 214 contigs. The majority of draft *Pelosinus* spp. genomes have one copy of the 16S rRNA gene predicted on a single contig (3, 4), except strain A11, which has two copies predicted (870 and 1,315 bp), and strain HCF1, which has five sequences (34, 127, 172, 228, and 1,218 bp) on five individual contigs (2). Two different versions of the 16S rRNA gene have been identified in strain UFO1 using PCR, one of which contained a 100-bp insertion and was not expressed under the conditions assayed (8).

A number of recent studies show that it is possible to generate complete microbial genome sequences using only single-molecule DNA sequencing technology (9–12). Repetitive DNA such as multiple ribosomal DNA operons are often one of the greatest barriers during the assembly process (13), and longer PacBio reads are useful in resolving these regions (14, 15).

This study employed the PacBio RSII sequencing system to generate a complete UFO1 genome sequence. The sequencing, assembly, and annotation methods have been described (14), except that this study employed three single-molecule real-time (SMRT) cells and used SMRT Analysis 2.1.1 and HGAP version 1 for assembly. The three SMRT cells generated 708,830,889 bp from 159,343 reads. The mean read length was 4,452 bp, and the longest was 24,144 bp. PacBio data assembled into a single contig, which gave a genome size of 5,114,828 bp for an estimated 139-fold genome coverage based on raw read values. The genome G+C content is 38.6%, 4,793 protein-encoding genes are predicted, and 15 copies of the 16S rRNA gene were detected. Four of the 16S rRNA genes were 1,647 bp and 100 bp longer than the remaining 11 copies, which is consistent with an earlier report indicating UFO1 intragenomic 16S rRNA heterogeneity due to a 100-bp insertion (8). Relationships between rRNA gene copy number, growth rate, and adaptation rate have been explored in the past (16–18). The complete UFO1 genome sequence and the high number of rRNA operons predicted may be useful for future comparative genomic studies and in assessing ecological strategies and structure in microbial communities.

### Nucleotide sequence accession numbers.

The genome sequence has been deposited at DDBJ/EMBL/GenBank under the accession number CP008852. The version described in this paper is the first version.
